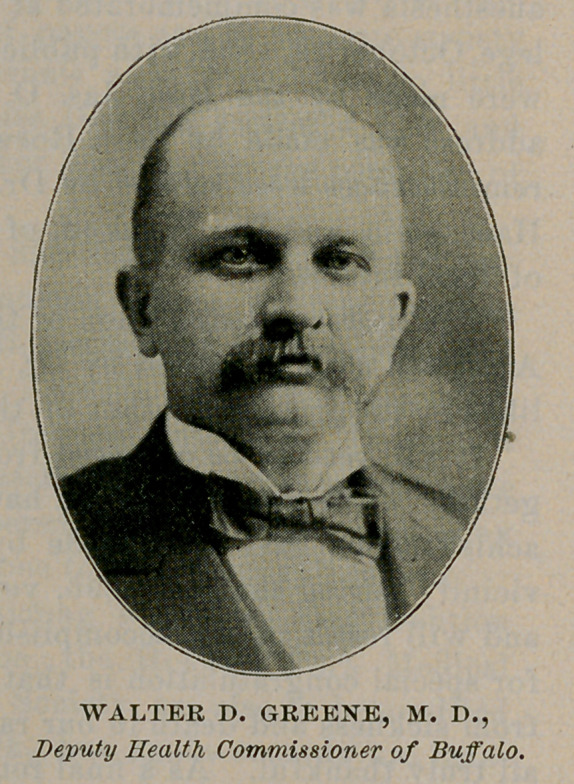# The Health Department—Its New Appointees

**Published:** 1897-01

**Authors:** 


### THE HEALTH DEPARTMENT—ITS NEW APPOINTEES.

ON WEDNESDAY, December 2, 1896, his Honor, Mayor Edgar B. Jewett, announced the appointment of Dr. Ernest Wende as health commissioner for a term of five years, beginning January 1, 1897, to succeed himself. The next day it was given out that Dr. Wende would appoint Dr. Walter D. Greene to be deputy health commissioner for the same term.

Scarcely a more gratifying circumstance could have happened in Buffalo than this action of the Mayor. The record Dr. Wende has made in the office during his term just ended is such as to justify on every account, and from every view-point, the action of the Mayor, and the fact that it has met with such uniform acquiescence is the strongest kind of an endorsement of the Mayor's action.

The fact is that the health department should be divorced from politics, and that appointments therein should be made with due regard to merit and efficient work, rather than to please any political party or faction thereof. Mayor Jewett has demonstrated his independence of action by resisting a pressure that must have been

ERNEST WENDE, M. D.,

*Health Commissioner of Buffalo.*

WALTER D. GREENE, M. D.,

*Deputy Health Commissioner of Buffalo.*

extreme. There are some men stupid enough to demand that a republican mayor must appoint only republicans to office, just as there are some others so stupid as to think that the worst kind of a democrat is better than the best kind of a republican. The Journal does not discuss politics, nor has it any sympathy with either of these extremes. The record Buffalo has obtained for good health and a low death-rate rendered it "dangerous to swap horses in the middle of the stream," as Mr. Lincoln was wont to say, hence, the only logical course was the reappointment of Dr. Wende.

Dr. Wende's action in promptly announcing the appointment of Dr. Greene was commendable. Dr. Greene's ability is unquestioned, and he has demonstrated his competency as the head of the department under the old charter. His many friends will be glad to see him again performing service in this department. We herewith present sketches of both these accomplished officers.

Ernest Wende, M.D., B.Sc., F.R.M.S., was born at Mill Grove, N. Y., on July 23, 1853. He began his education in his native town and after attending the district school he entered the Clarence Academy and later was graduated from School No. 10 in this city. He entered the Buffalo High School and, after graduating, spent a year at West Point. By teaching school for a few terms at South Newstead and in the town of Alden he managed to earn enough money to enter the University of Buffalo, where he graduated with honors in 1878. Then he was elected school commissioner for three years and afterward took a post-graduate course at the College of Physicians and Surgeons, New York. He finished the course in 1882 and entered the University of Pennsylvania. In 1884 he was graduated, taking honors of the first class. While there he took the George B. Wood alumni prize. There were forty-three competitors. In 1885 he received the degree of Bachelor of Science from this college.

After he was graduated from the University of Pennsylvania, Dr. Wende went to Berlin and Vienna. He was a student in Prof. Virchow's private laboratory, making a special study of pathology. He also took a special course in bacteriology under Prof. Koch. He was assistant to Prof. Behrend in his clinic on diseases of the skin. He also pursued the study of skin diseases under Profs. Köbner, Lewin and Lassar at Berlin. At Vienna he continued his studies in dermatology under Profs. Kaposi, Neumann, Ullmann and Hebra. He returned to Buffalo in 1886, since making a special practice of skin diseases.

Soon after his return he was appointed clinical professor of diseases of the skin in the University of Buffalo. When Prof. Kellicott was called to the University of Ohio he succeeded him as professor of botany and microscopy in the department of pharmacy, University of Buffalo.

In 1892 Dr. Wende was appointed health commissioner by Mayor Bishop, the first to serve under the new charter.

Dr. Wende is a member of the Royal Microscopical Society of London; vice-president of the American Public Health Association; member of the business committee of the Medical Society of the State of New York, 1897; Fellow of the American Electro-Therapeutic Association; Fellow of the American Microscopical

Society ; member of the Medical Society of the County of Erie ; member of the Buffalo Society of Natural Sciences ; member of the Buffalo Academy of Medicine, of the Buffalo Medical Club and of the Buffalo Medical Union, and one of the associate editors of the BUFFALO MEDICAL JOURNAL.

Walter D. Greene, M.D., was born in Starksboro, Vt., in 1853. He first went to the country school near his home, and then attended Union Seminary at Union Springs, N. Y. He was graduated in 1871. Later he entered the medical department of the University of Buffalo, where he was graduated in 1876. He then became attached to the Rochester City Hospital.

He began to practise in Mendon, Monroe county, in 1878. Two years afterwards he opened an office on Elk street in this city. He was made a district health physician in 1882, and in 1889 was appointed city health physician. In 1891 he took the chair of hygiene and preventive medicine in the Niagara University Medical College. Dr. Greene is a member of the Medical Society of the County of Erie, of the New York State Medical Association, the Buffalo Academy of Medicine and the Buffalo Club, a director of the Acacia Club and of the Masonic Life Association of Western New York.

## Figures and Tables

**Figure f1:**
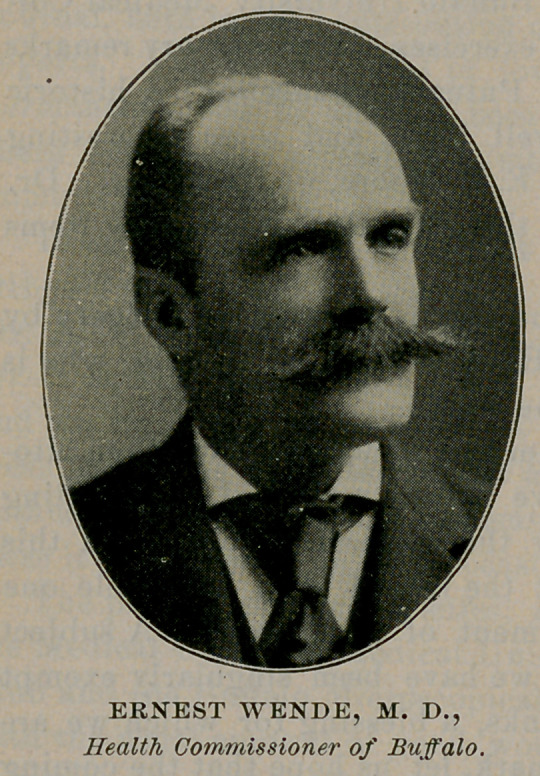


**Figure f2:**